# Does therapeutic hypothermia during extracorporeal cardiopulmonary resuscitation preserve cardiac function?

**DOI:** 10.1186/s12967-016-1099-y

**Published:** 2016-12-20

**Authors:** Harald A. Bergan, Per S. Halvorsen, Helge Skulstad, Erik Fosse, Jan F. Bugge

**Affiliations:** 1 Division of Emergencies and Critical Care, Department of Research and Development, Oslo University Hospital, Oslo, Norway; 2 Faculty of Medicine, Institute of Clinical Medicine, University of Oslo, Oslo, Norway; 3 The Intervention Centre, Rikshospitalet, Oslo University Hospital, Oslo, Norway; 4 Department of Cardiology, Rikshospitalet, Oslo University Hospital, Oslo, Norway

**Keywords:** Cardiac function, Cardiopulmonary resuscitation, Extracorporeal circulation, Extracorporeal membrane oxygenation, Therapeutic hypothermia

## Abstract

**Background:**

Extracorporeal cardiopulmonary resuscitation (E-CPR) is increasingly used as a rescue method in the management of cardiac arrest and provides the opportunity to rapidly induce therapeutic hypothermia. The survival after a cardiac arrest is related to post-arrest cardiac function, and the application of therapeutic hypothermia post-arrest is hypothesized to improve cardiac outcome. The present animal study compares normothermic and hypothermic E-CPR considering resuscitation success, post-arrest left ventricular function and magnitude of myocardial injury.

**Methods:**

After a 15-min untreated ventricular fibrillation, the pigs (n = 20) were randomized to either normothermic (38 °C) or hypothermic (32–33 °C) E-CPR. Defibrillation terminated ventricular fibrillation after 5 min of E-CPR, and extracorporeal support continued for 2 h, followed by warming, weaning and a stabilization period. Magnetic resonance imaging and left ventricle pressure measurements were used to assess left ventricular function pre-arrest and 5 h post-arrest. Myocardial injury was estimated by serum concentrations of cardiac TroponinT and Aspartate transaminase (ASAT).

**Results:**

E-CPR resuscitated all animals and the hypothermic strategy induced therapeutic hypothermia within minutes without impairment of the resuscitation success rate. All animals suffered a severe global systolic left ventricular dysfunction post-arrest with 50–70% reductions in stroke volume, ejection fraction, wall thickening, strain and mitral annular plane systolic excursion. Serum concentrations of cardiac TroponinT and ASAT increased considerably post-arrest. No significant differences were found between the two groups.

**Conclusions:**

Two-hour therapeutic hypothermia during E-CPR offers an equal resuscitation success rate, but does not preserve the post-arrest cardiac function nor reduce the magnitude of myocardial injury, compared to normothermic E-CPR.

*Trial registration* FOTS 4611/13 registered 25 October 2012

**Electronic supplementary material:**

The online version of this article (doi:10.1186/s12967-016-1099-y) contains supplementary material, which is available to authorized users.

## Background

Survival after cardiac arrest is greatly influenced by early post-arrest cardiac function [1, 2]. Hence, cardiopulmonary resuscitation (CPR) strategies that preserve post-arrest cardiac function may improve outcome. Extracorporeal CPR (E-CPR) by veno-arterial extracorporeal membrane oxygenation (ECMO) is increasingly used when standard CPR fails. Promising results have been reported by using E-CPR as a rescue method within brief timeframes for selected cases [3–6]. Therapeutic hypothermia (HT, 32–34 °C) is widely used for patients resuscitated from cardiac arrest as it is believed to exhibit cardiovascular [7, 8] and neurological benefits [9–11].

To achieve cardiac benefit from HT the importance of early and rapid cooling has been emphasized in experimental studies [12–14]. E-CPR provides the opportunity to rapidly induce HT, but whether hypothermic E-CPR preserves post-arrest cardiac function and hence improves outcome, is not known.

A severe cardiac dysfunction following normothermic E-CPR is recently demonstrated in pigs [15]. The present study aimed to investigate if HT during E-CPR improves cardiac outcome early post-arrest. We hypothesized that hypothermic E-CPR offers an equal resuscitation success rate, but with a better preserved post-arrest left ventricular (LV) function and less myocardial injury compared to normothermic E-CPR.

## Methods

### Design

A prospective controlled block-randomized animal study was completed to compare a normothermic (38-ECPR, n = 10) and a hypothermic (32-ECPR, n = 10) E-CPR group.

### Animal welfare

The experimental protocol was approved by the Norwegian National Animal Research Authority and the animal experiments were performed in accordance with the European Convention for the Protection of Vertebrate Animals used for Experimental and Other Scientific Purposes (European Council, ETS No. 170). Detailed information according to the ARRIVE guidelines is presented in Additional file 1: Table S1 [16]. With respect to animal welfare`s 3R-principle, eight 38-ECPR animals were included in a separate methodological study demonstrating E-CPR associated post-arrest LV dysfunction by cardiac magnetic resonance imaging (MRI) per se [15].

### Animal preparation

The animal preparation included premedication in the pig enclosure and total intravenous anaesthesia in the operating theatre, mechanical ventilation and a succeeding surgical tracheotomy and placements of intravascular catheters and ECMO-cannulas as recently described [15]. Ringer’s acetate solution was infused at 10 ml/kg/h.

## Experimental protocol

### Baseline assessments

After the preparation and a following 30-min stabilization period, baseline cardiac MRI (Philips Achieva 3 Tesla, Philips Medical Systems, DA Best, Netherland) and haemodynamic measurements of LV function (MPR-500, Millar Instruments, Houston, TX, USA) were obtained. Arterial and mixed venous blood samples were analyzed (ABL 800 Flex, Bergman Diagnostika, Kjeller, Norway) and serum concentrations of cardiac Troponin T (cTnT) and Aspartate aminotransferase (ASAT) were measured.

### ECMO and cardiac arrest

After baseline assessments, the pig was connected to a Ringer’s acetate-primed femoro-jugular veno-arterial ECMO circuit (Biopump + BPX-80/Affinity NT/Biomedicus 550, Medtronic Inc, Minneapolis, MN, USA) featuring an oxygen/air mixer (Sechrist Model 20090, Sechrist Industries, Anaheim, CA, USA) to adjust sweep gas oxygen content and sweep gas flow rate. A connected heat-exchanger (Stöckert Heater-Cooler System 3T, Sorin Group, Milano, Italy) enabled animal blood temperature control.

After an intravenous injection of 2 mg/kg heparin, an electrical stimulator connected to a right ventricular pacing lead (Qstim 5Fr, VascoMed GmbH, Binzen, Germany) induced ventricular fibrillation (VF), confirmed by ECG shape and aortic blood pressure drop.

### E-CPR

After 15 min of untreated VF the animals received either normothermic [pulmonary artery blood temperature 38.0 °C (normothermia in the pig)] or hypothermic (32.0–33.0 °C) E-CPR at maximum ECMO blood flow rate with a 100% oxygen sweep gas set at the same flow rate as the ECMO blood flow rate.

HT in the 32-ECPR group was achieved using 20.0 °C priming solution with later adjustments at the heat-exchanger. The heat-exchanger thermostat at 38.0 °C ensured normothermia in the 38-ECPR group.

After 5 min of E-CPR 360 Joule monofasic defibrillations (CodeMaster XL + Hewlett Pachard, Lexington, KY, USA) were provided until regain of spontaneous cardiac beating (ROSB) with extracorporeal support continuing at unchanged blood flow rate and temperature target for 120 min. In the 32-ECPR group a 30-min warming period followed, whereas a corresponding 30-min continued run at 38.0 °C was provided in the 38-ECPR group.

The mean aortic blood pressure (MAP) target (≥50 mmHg) and pulse-pressure target (≥15 mmHg) after ROSB were met using repeated 10–25 µg adrenaline (epinephrine) intravenously followed by dobutamine infusion if needed [17].

### Weaning from ECMO

After the 120 + 30-min extracorporal support, with all animals being normothermic, a step-wise separation from ECMO (weaning) was completed during a 60-min period, and the animals were allowed to stabilize after weaning for another 60 min before the post-arrest assessments.

### Post-arrest assessments

At 285 min post-arrest LV function was re-assessed by LV pressure measurements and MRI. Finally, a second arterial and mixed venous blood sample were analyzed and blood samples for cTnT and ASAT measurements were collected, before the pig was euthanized.

## Measurements

### MRI and haemodynamic measurements

**Fig. 1:**
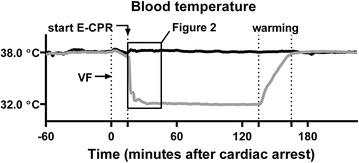
Blood temperature. After a 15-min untreated ventricular fibrillation (VF) the animals were randomized to either normothermic (38 °C) or hypothermic (32–33 °C) extracorporeal cardiopulmonary resuscitation (E-CPR). A 30-min rewarming period (between *dotted lines*) started in the hypothermic group after 120 min of extracorporeal support. Blood temperature (mean, *connecting line*) was recorded every 30 s

**Fig. 2:**
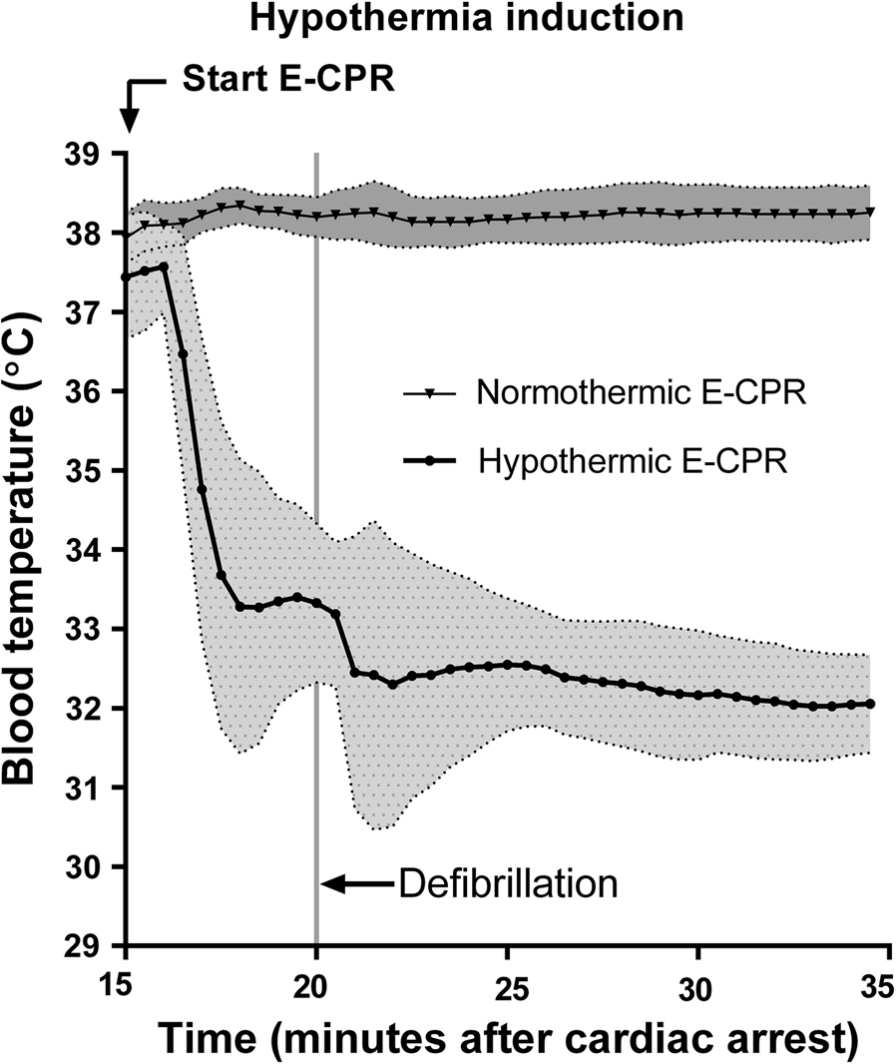
Induction of therapeutic hypothermia. The temperature dropped after initiation of hypothermic extracorporeal cardiopulmonary resuscitation (E-CPR) to the targeted 32–33 °C within the 1st min after defibrillation. Blood temperature [mean (*connecting line*) ± standard deviation (*shaded*)] was recorded every 30 s

**Fig. 3:**
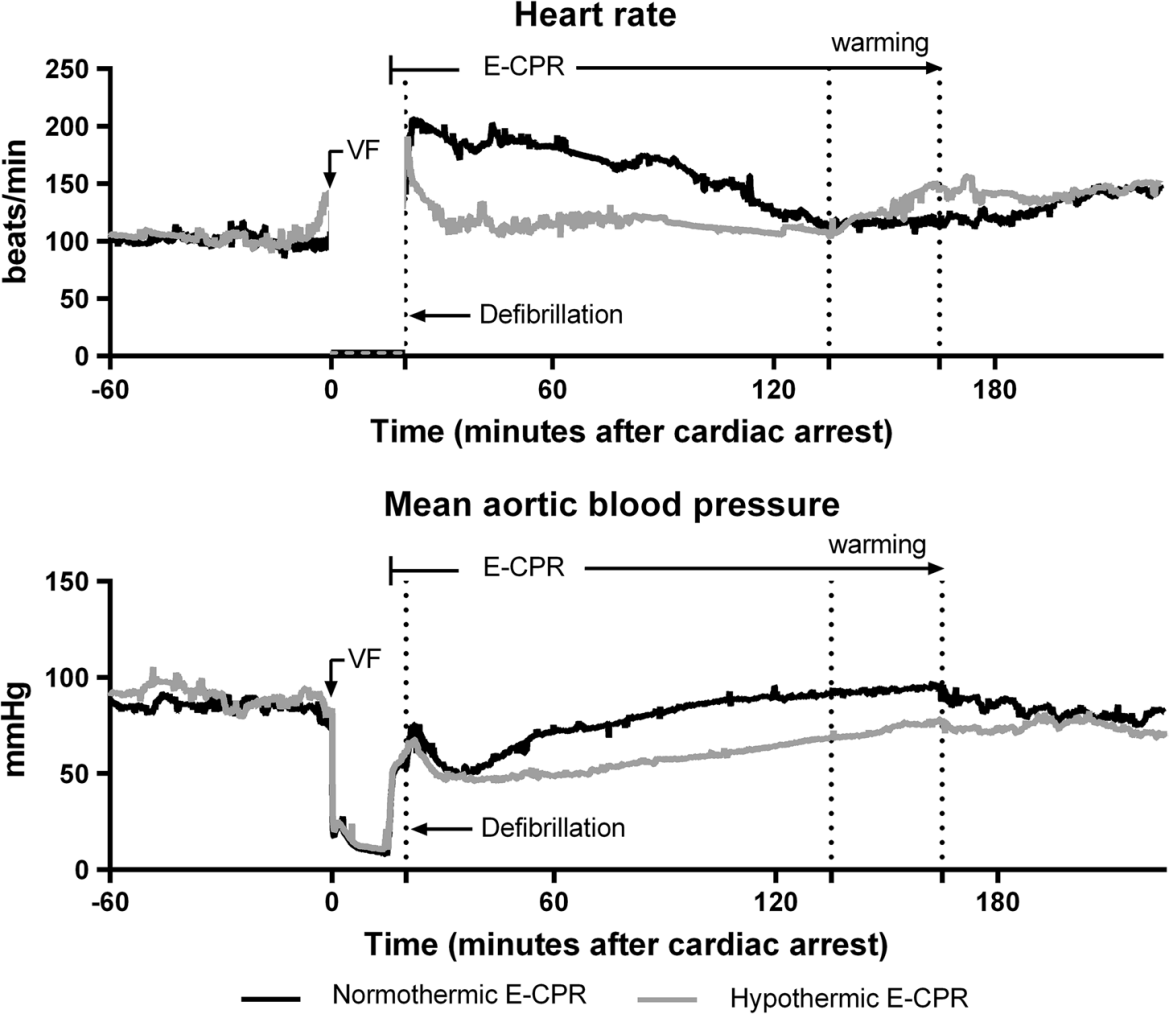
Heart rate and mean aortic blood pressure. Continuous measurements (mean, *connecting line*) of heart rate and mean aortic blood pressure in the two different treatment groups. *VF* ventricular fibrillation

Magnetic resonance imaging was used to assess LV volumes and function (end-systolic volume (ESV), end-diastolic volume (EDV), stroke-volume (SV = EDV–ESV), ejection fraction (EF  =  SV/EDV), cardiac output [CO = SV heart rate (HR)] mid LV radial wall thickening, mitral annular plane systolic excursion (MAPSE), and peak global systolic LV circumferential strain) together with LV pressure measurements (maximum systolic LV pressure (LVP_max_), maximum positive and negative first time derivate of LV pressure (dP/dt_max_ and dP/dt_min_), end-diastolic LV pressure (EDP), end-systolic LV pressure (ESP), isovolumetric relaxation constant (tau), and arterial elastance (Ea  =  ESP/SV)) [15, 18]. Continuous measurements of blood temperature (Figs. 1, 2), HR and MAP (Fig. 3) outside the MRI were recorded throughout the experiments.

### cTnT and ASAT

The serum concentration measurements of cTnT and ASAT pre-arrest and 6 h post-arrest were performed by an electro-chemiluminescence immunoassay (Troponin T hs, Roche Diagnostics, Rotkreuz, Switzerland) and by an UV-test with pyridoxal phosphate activation (ASAT, Roche Diagnostics) using an automated clinical chemistry analyzer (Modular analytical platform, module E170 and P800, Roche Diagnostics) to estimate myocardial injury.

### Myocardial tissue staining

Eleven (38-ECPR n = 5, 32-ECPR n = 6) hearts excised immediate post-mortem were sliced and stained in 1% triphenyl tetrazolium chloride (TTC, Sigma Chemical Co., St. Louis, MO, USA) in phosphate buffer and examined for myocardial infarction [19, 20].

## Statistical analysis

Statistical analyses were made using Graphpad prism 6.04 (GraphPad Software, La Jolla, CA, USA). Data are reported as mean ± standard deviation if not otherwise stated. The statistical significance level α was set to 0.05 and power 1 −β to 0.80.

The study sample size was estimated by a prospective power analysis. The least detectable difference considered as clinically significant between cardiac function variables was 15% of baseline values. Cardiac function variability in range of 5–15% of baseline values in pilot experiments made a calculated sample size of 20 necessary to achieve the desired power.

A paired two-tailed Student's *t* test (t) was used to compare baseline and post-arrest measurements within each treatment group, and an unpaired two sample *t*-test (T) was used to compare post-arrest measurements between the two different treatment groups. Alternatively, a two-tailed Mann–Whitney test (MW) of group differences with exact p-value was used for data not normally distributed.

## Results

Animal weight (49.2 ± 2.8 kg), preparation time (122 ± 22 min) and cardiac MRI scan time (63 ± 15 min) were similar in the two groups. Neither activated clotting-time prior to cardiac arrest (330 ± 76 s) nor ECMO circuit priming volume (544 ± 22 ml) differed between the two groups, and the experiment durations were also similar, averaging 762 ± 63 min (32-ECPR 777 ± 58 min vs. 38-ECPR 745 ± 67 min; T, p = 0.27).

One animal in the 38-ECPR group was euthanized after ROSB, and was thus excluded from further analyses, because MAP could not be sustained as dictated by the protocol, due to ECMO venous cannula malfunctioning.

At initiation of VF the blood temperature was 38.0 ± 0.2 °C and was maintained at this level in the 38-ECPR group (Fig. 1). In the 32-ECPR group the temperature quickly dropped after initiation of hypothermic E-CPR and was 33.3 ± 1.0 °C at the time of defibrillation (Fig. 2). It further dropped to the targeted 32–33 °C within the 1st min after defibrillation (5.25 ± 4.8 min from start E-CPR) and was kept stable at this level until warming.

### Defibrillation

All animals were successfully defibrillated by a median of 1 shock (range 1–6) with no significant differences between the groups (MW, p = 0.99). After ROSB three animals in the 38-ECPR group spontaneously had a second VF, and immediately received a median of 1 (range 1–3) additional defibrillations. No additional defibrillations were needed in the 32-ECPR group (MW, p = 0.21).

MAP at the time of defibrillation was 54 ± 9 mmHg in the 38-ECPR group and 60 ± 5 mmHg in the 32-ECPR group (T, p = 0.082) (Fig. 3) with an ECMO blood flow rate of 4.6 ± 0.1 l/min and 4.4 ± 0.2 l/min in the two groups, respectively (T, p = 0.055).

### Inotropes

The adrenaline dosage [median (range)] was 100 µg (0–340 µg) in the 38-ECPR group vs. 183 µg (50–1700 µg) in the 32-ECPR group (MW, p = 0.074). The dobutamine requirements differed between the groups [38-ECPR dobutamine [median (range)] = 15.8 mg (0–31.2 mg) vs. 32-ECPR dobutamine = 36.2 mg (10–93.6 mg)] (MW, p = 0.0076), but the dosages after ECMO weaning to the end of the experiments were not significantly different; four of nine animals in the 38-ECPR group and five of ten in the 32-ECPR group received dobutamine dosages [median (range)] of 2 mg (0.5–2.2) and 2.6 mg (0.01–5 mg), respectively, after weaning (MW, p = 0.40).

### Haemodynamic measurements and blood gas analyses

HR increased similarly post-arrest in both groups (Table 1), limiting the reductions in CO. The increased Ea post-arrest did not differ between the groups, and the dP/dt_max_, dP/dt_min_, EDP, EDP/EDV relationship, and tau did not change significantly from baseline values in either group.

**Table 1:** Haemodynamic measurements and blood gas analyses

Variable	Normothermic E-CPR group		Hypothermic E-CPR group		Post-arrest, difference between groups	
	Baseline	Post-arrest	Baseline	Post-arrest	MD (95% CI)	p value
a. Haemodynamic measurements						
HR (beats/min)	88 ± 23	152 ± 31*	101 ± 26	148 ± 33*	−4 (−35 27)	p = 0.79
MAP (mmHg)	84.2 ± 12.0	73.6 ± 16.1	93.1 ± 14.4	65.6 ± 14.1*	−8.1 (−22.6 6.5)	p = 0.26
CVP (mmHg)	5.7 ± 2.8	6.1 ± 3.1	6.9 ± 1.6	8.7 ± 2.9	2.6 (−0.3 5.5)	p = 0.078
LVP_max_ (mmHg)	99.6 ± 7.0	91.0 ± 13.5	107.7 ± 13.2	86.4 ± 14.8*	−4.6 (−18.4 9.2)	p = 0.49
EDP (mmHg)	14.0 ± 4.7	14.9 ± 2.4	12.9 ± 3.7	11.7 ± 3.7	−3.2 (−6.3 −0.1)	p = 0.041
dP/dtmax (mmHg/s)	1427 ± 207	1963 ± 557	1786 ± 414	2398 ± 1466	435 (−663 1533)	p = 0.41
dP/dtmin (mmHg/s)	−2112 ± 270	−1683 ± 404	−2225 ± 315	−1792 ± 832	−109 (−755 537)	p = 0.73
tau (ms)	32.4 ± 3.0	33.3 ± 6.8	32.3 ± 3.2	28.5 ± 8.3	0.68 (−5.7 7.1)	p = 0.19
EDP/EDV (mmHg/ml)	0.14 ± 0.04	0.16 ± 0.03	0.14 ± 0.04	0.14 ± 0.04	−0.02 (−0.06 0.01)	p = 0.15
Ea (mmHg/ml)	1.08 ± 0.25	1.65 ± 0.44*	1.16 ± 0.35	1.68 ± 0.72*	0.03 (−0.56 0.62)	p = 0.91
b. Blood gas analyses						
Hb (g/dl)	8.3 ± 0.9	8.1 ± 0.7*	8.7 ± 0.8	7.8 ± 1.1*	−0.3 (−1.3 0.6)	p = 0.45
PaO_2_ (kPa)	22.5 ± 1.9	20.6 ± 3.7	22.6 ± 1.4	21.5 ± 2.4	0.90 (−2.1 −3.9)	p = 0.53
PaCO_2_ (kPa)	5.4 ± 0.7	5.4 ± 0.3	4.9 ± 0.4	4.9 ± 0.4	−0.45 (−0.82 −0.08)	p = 0.019
pH	7.49 ± 0.04	7.47 ± 0.03	7.51 ± 0.05	7.48 ± 0.05	−0.01 (−0.03 0.06)	p = 0.63
BaseExcess (mmol/l)	6.77 ± 1.15	4.97 ± 1.82*	6.23 ± 2.38	3.56 ± 2.12*	−1.41 (−3.39 0.56)	p = 0.15
Lactate (mmol/l)	0.85 ± 0.19	1.28 ± 0.54*	1.34 ± 0.85	2.8 ± 1.57*	1.52 (0.36 2.68)	p = 0.013
Ca^2+^ (mmol/l)	1.30 ± 0.06	1.27 ± 0.06	1.25 ± 0.06	1.27 ± 0.06	0.003 (−0.06 0.06)	p = 0.91
SvO_2_ (%)	65.7 ± 14.8	42.4 ± 8.7*	68.4 ± 7.4	42.3 ± 13.4*	−0.2 (−11.2 −10.9)	p = 0.98

Values are expressed as mean ± standard deviation. Comparison post-arrest to baseline within group by paired student’s *t*-test

Post-arrest comparison of groups by unpaired two-sample student’s *t*-test. *MD* mean difference, *CI* 95% confidence interval

*HR* heart rate, *MAP* mean aortic blood pressure, *CVP* central venous pressure, *LVP*_*max*_ systolic left ventricular pressure maximum, *EDP* end-diastolic pressure, *dP/dt*_*max*_ maximum left ventricular pressure first time derivate, *dP/dt*_*min*_ minimum left ventricular pressure first time derivate, *tau* isovolumetric relaxation constant, *Ea* arterial elastance, *S*_*V*_ *O*_*2*_ mixed venous oxygen saturation

* p ≤ 0.05

Mixed venous oxygen saturation dropped considerably and group-similarly post-arrest (Table 1). A small decrease in haemoglobin content, base excess and pH was also measured post-arrest, with an accompanying increase in arterial lactate.

### Cardiac MRI

**Fig. 4:**
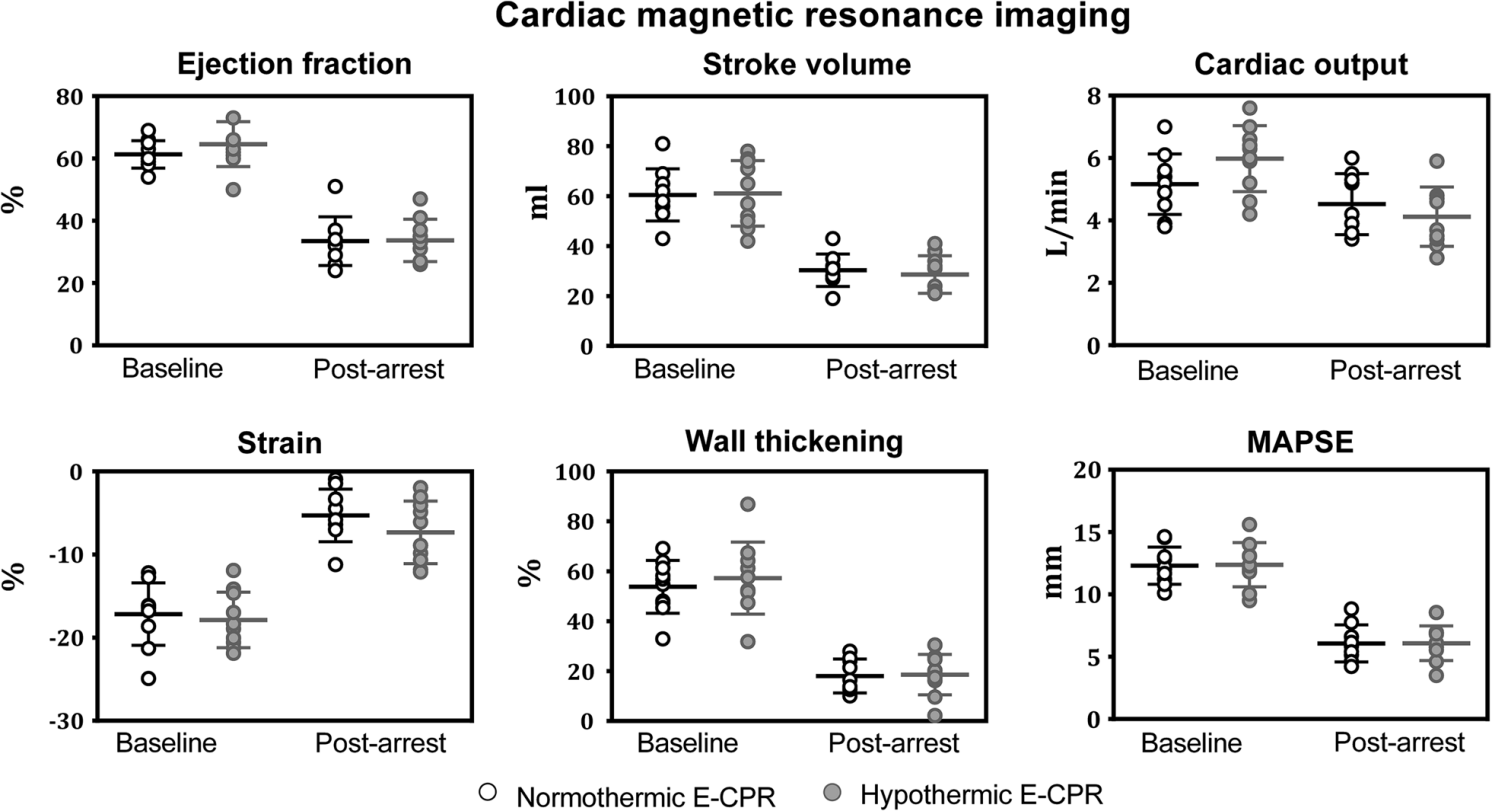
Cardiac MRI measurements. The systolic left ventricle function variables did not differ between the normothermic and the hypothermic E-CPR group at baseline or post-arrest. Strain, peak global systolic left ventricular circumferential strain; Wall thickening, radial mid left ventricular wall thickening; MAPSE, mitral annular plain systolic excursion. Line at mean ± standard deviation (*short line*)

In both groups the LV function was severely affected post-arrest (Fig. 4). EF decreased from 61 ± 4% to 33 ± 8% (t, p < 0.001) and SV decreased from 61 ± 10 to 30 ± 6 ml (t, p < 0.001) in the 38-ECPR group. In the 32-ECPR group EF decreased from 65 ± 7 to 34 ± 7% (t, p < 0.001) (T, p = 0.94) and SV decreased from 61 ± 13 to 29 ± 8 ml (t, p < 0.001) (T, p = 0.60). In the 38-ECPR group the lowered EF was only related to the increase in ESV from 38 ± 6 to 61 ± 14 ml (t, p = 0.001) as the EDV was maintained at 92 ± 14 ml from a baseline EDV value of 99 ± 13 ml (t, p = 0.44). There was a similar increase in ESV in the 32-ECPR group from 34 ± 10 to 56 ± 11 ml (t, p < 0.001). In this group, however, EDV was moderately reduced from a baseline EDV value of 95 ± 18 ml to post-arrest EDV 85 ± 14 ml (t, p = 0.0044). Despite severe tachycardia CO was reduced in most animals post-arrest (six of nine in the 38-ECPR group; nine of ten in the 32-ECPR group), but CO did not significantly differ between the two groups post-arrest; 38-ECPR CO = 4.5 ± 1.0 l/min vs. 32-ECPR CO = 4.1 ± 0.9 l/min (T, p = 0.38).

Consistent with the reduced SV the strain decreased group-alike; from −17 ± 4 to −5 ± 3% (t, p < 0.001) in the 38-ECPR group and from −18 ± 3 to −7 ± 4% (t, p < 0.001) in the 32-ECPR group (T, p = 0.22). MAPSE in the 38-ECPR group was reduced from 12 ± 1 to 6 ± 1 mm (t, p < 0.001) and likewise in the 32-ECPR group from 12 ± 2 to 6 ± 1 mm (t, p < 0.001) (T, p = 0.98). Correspondingly, the wall thickening in the 38-ECPR group was severely affected by a reduction from 54 ± 11 to 18 ± 7% (t, p < 0.001). A similar reduction from 57 ± 14 to 13 ± 24% (t, p < 0.001) was found in the 32-ECPR group (T, p = 0.87).

### cTnT and ASAT

The serum concentrations of cTnT and ASAT remained minimal after the surgical preparation (38-ECPR cTnT (median (range)) = 13 (10–32) ng/L vs. 32-ECPR cTnT= 16 (7–28) ng/L; 38-ECPR ASAT = 27 (18–33) U/L vs. 32-ECPR ASAT = 28 (18–31)) U/L, but increased considerably post-arrest without significant differences between the groups; 38-ECPR cTnT = 2295 (782–5203) ng/L vs. 32-ECPR cTnT = 2452 (925–5475) ng/L (MW, p = 0.44) and 38-ECPR ASAT = 123 (61–1630) U/L vs. 32-ECPR ASAT = 266 (112–969) U/L (MW, p = 0.13).

### TTC staining post mortem

The assessment of myocardial infarction by TTC staining post mortem did not demonstrate regional infarctions.

## Discussion

In the present controlled animal study comparing hypothermic and normothermic E-CPR considering resuscitation success, post-arrest LV function, and myocardial injury, surprisingly, and contrary to our hypothesis, no beneficial effects of HT during E-CPR could be demonstrated.

A better preserved LV function would be desirable in resuscitated patients as post-arrest cardiac function is related to patient survival. In the present study, HT during E-CPR was hypothesized as being beneficial because HT has demonstrated cardioprotective effects in various animal studies with regional myocardial ischaemia (i.e. coronary occlusion) [21, 22].

The best strategy of E-CPR to preserve early post-arrest cardiac function is not known and no guidelines exist to assist clinicians deciding on an E-CPR strategy for patients in refractory cardiac arrest. To date HT is not recommended as a cardioprotective intervention in patients with acute myocardial infarction without associated cardiac arrest [22, 23]. In resuscitated patients, however, HT is an established treatment due to neurological benefits, irrespective of any cardioprotection [9–11]. Whole body cooling targeting 32–36 °C is the latest recommendation (preferably a constant temperature in this range) and a HT induction time-frame of 4–6 h post-arrest is usually accepted [24]. A delay of several hours from resuscitation to target temperature may exclude a cardioprotective effect of hypothermia per se. HT may nevertheless be favorable to the cardiovascular function as it may reduce cardiac work load as a consequence of reduced whole body metabolism during HT [25].

Correct timing of HT has been emphasized in recent years as HT is claimed to be cardioprotective only if induced either shortly before or at the time point of myocardial reperfusion [12, 13, 26, 27]. Maeng and co-workers found that HT induced at the time of coronary reperfusion did not reduce myocardial infarct size in pigs [28]. Despite the efficient HT induction, the present study did not demonstrate cardioprotection by hypothermic E-CPR (i.e. reperfusion) of the fibrillating heart. Cooling the myocardium prior to myocardial reperfusion may thus be a crucial procedure, but effort is needed to achieve hypothermia this early in the clinical coronary occlusion scenario [29, 30]. Correspondingly, a rationale for cardioprotective intra-arrest HT (i.e. HT induced before ROSB) exists in cardiac arrested patients, and is supported by animal studies [13, 31, 32], but the suggested post-arrest cardiac function benefits are not specifically investigated in humans. If interventions to reperfuse the myocardium are postponed until HT is established, the harm of prolonged ischaemia may cancel any benefits of HT. This issue could be further investigated experimentally using topical cooling of the arrested heart prior to reperfusion.

Two-hour HT duration was investigated as brief cooling has been sufficient to achieve cardioprotective effects in previous animal studies [12] and 3-h cooling has made myocardial damage worse [33]. Whether a longer HT duration would be beneficial in our study is not known, but cannot be excluded. On the other hand, extracorporeal circulatory support by ECMO is not without complications and side effects, and clinical practice is to wean as soon as the heart is capable to independently handle the circulation.

Compared to our study, rewarming of patients after cardiac arrest is slow (0.3–0.5 °C/h), tailored for neuroprotection [24]. In cardiac surgery, however, a quick rewarming is well tolerated by the heart even after long-lasting cardioplegic arrest, and we have no indications that a longer and slower rewarming period would have influenced our results.

### Inotropes

Inotropic support is regularly used during VA–ECMO to sustain aortic ejections with a sufficient pulse-pressure to avoid LV distention and failure. The increased dobutamine requirements observed during HT may be related to altered pharmacologic properties of dobutamine with a reduced effect [34] as supported by the similar requirements in the two groups after rewarming. HT also affects LV function, causing slower LV contraction and relaxation velocities [35], and may thus increase the need for inotropic stimulation to reach preset targets. The optimal MAP target during HT is not known, and could possibly differ from MAP target at normothermia, but for comparison, they were set at the same level.

### LV function

In the present study, the 50–70% reductions in SV, EF, wall thickening, strain and MAPSE were consistent, demonstrating a severe global systolic LV dysfunction post-arrest with uniform impairments in all directions of systolic LV motion, and with no differences between the two treatment groups.

The diastolic LV function assessed by dP/dt_min_, tau and EDP/EDV relationship was preserved in both groups post-arrest, and neither EDV (preload) nor Ea (afterload) differed between the two groups.

### Myocardial injury evaluation

The post-arrest LV dysfunction indicated a severe and global myocardial injury as was confirmed by the considerable increase in serum concentrations of cTnT and ASAT in both groups. A global injury without distinguished regional areas was also confirmed by the TTC assessment, as no regional infarctions could be demonstrated, excluding coronary thrombus or embolic complications.

The success rate of resuscitation by hypothermic E-CPR was not inferior to the normothermic strategy, and neither LV function nor myocardial injury was exacerbated. E-CPR initiated by a room tempered ECMO may be convenient, as a normothermic ECMO (or a circuitry heating device) will not always be available in clinical emergency settings that may include emergency rooms, ambulance transfers and even pre-hospital use.

### Limitations

The clinical scenario of E-CPR differs from a controlled animal experiment as the period of no-flow is usually short (preferably <5 min) and the patient is cannulated at ongoing CPR in a low-flow period of varying duration. In the present study, a healthy pig heart suffered an electrically induced VF and the total ischemic insult was prepared to be substantial and consistent to assure a significant post-arrest cardiac dysfunction and injury that could be compared between the two different treatment groups. The duration of no-flow thus exceeded usual clinical limits, and the low-flow period was bypassed.

## Conclusions

E-CPR is an effective resuscitation technique for prolonged cardiac arrest that may rapidly induce HT. In the present animal study, 2-h HT during E-CPR offered an equal resuscitation success rate, but did not preserve the post-arrest cardiac function nor reduce the magnitude of myocardial injury, compared to normothermic E-CPR.

## Additional file

**Additional file 1: Table S1.** Detailed information on the experimental animals according to ARRIVE^*^ guidelines.

## Abbreviations

ASAT: aspartate transaminase
CO: cardiac output
CPR: cardiopulmonary resuscitation
CVP: central venous pressure
dP/dt _max_ and dP/dt _min_: maximum and minimum LVP first time derivates
Ea: arterial elastance
ECMO: extracorporeal membrane oxygenation
E-CPR: extracorporeal cardiopulmonary resuscitation
EDP: end-diastolic pressure
EDV: end-diastolic volume
ESP: end-systolic pressure
HR: heart rate
LVP: left ventricular pressure
LVP _max_: systolic left ventricular pressure maximum
MAP: mean aortic pressure
MRI: magnetic resonance imaging
ROSB: regain of spontaneous cardiac beating
SV: stroke volume
S_V_O_2_: mixed venous oxygen saturation
tau: isovolumetric relaxation constant
cTnT: cardiac Troponin T
